# New Developments in the Pharmacological Treatment of Hypertension: Dead-End or a Glimmer at the Horizon?

**DOI:** 10.1007/s11906-015-0557-x

**Published:** 2015-04-19

**Authors:** Ludovit Paulis, Romana Rajkovicova, Fedor Simko

**Affiliations:** Institute of Pathophysiology, Faculty of Medicine, Comenius University, Sasinkova 4, 81108 Bratislava, Slovak Republic; Institute of Normal and Pathological Physiology, Slovak Academy of Sciences, Sienkiewiczova 1, 81371 Bratislava, Slovak Republic; Institute of Experimental Endocrinology, Slovak Academy of Sciences, Vlárska 3, 83306 Bratislava, Slovak Republic

**Keywords:** Hypertension, Neprilysin, Renin-angiotensin-aldosterone system, Angiotensin peptides, Angiotensin AT2 receptor, Drug combination

## Abstract

Arterial hypertension is the most prevalent controllable disease world-wide. Yet, we still need to further improve blood pressure control, deal with resistant hypertension, and we hope to reduce risk “beyond blood pressure.” The number of candidate molecules aspiring for these aims is constantly declining. The new possible approaches to combat high blood pressure include neprilysin/neutral endopeptidase (NEP) inhibition, particularly when combined with an angiotensin receptor blockade (such as the ARNI, LCZ696), phosphodiesterase 5 (PDE5) inhibition (KD027/Slx-2101), natriuretic agents (PL3994), or a long-lasting vasointestinal peptide (VIP) analogue (PB1046). Other options exploit the protective arm of the renin-angiotensin-aldosterone system by stimulating the angiotensin AT2 receptor (compound 21), the Mas receptor (AVE-0991), or the angiotensin converting enzyme 2. Finally, we review the possibilities how to optimize the use of the available treatment options by using drug combinations or by tailoring therapy to each patient’s angiotensin peptide profile.

## Introduction

Arterial hypertension is a wide-spread but controllable disease affecting as much as 30–45 % of general population [1]. Despite the broad spectrum of the already available pharmacological (as well as nonpharmacological) means for blood pressure control, there is no evidence for a change in average blood pressure values over the past decades [1]. Moreover, the rate of stroke (as an indirect indicator of blood pressure levels in the population) tends to increase in eastern European countries [2]. Thus, there is an obvious medical demand for novel approaches to treat high blood pressure.

At the first glance, there is a huge interest in the development of novel pharmacological agents. The latest available *Pharmaceutical Research and Manufacturers of America* (PhRMA) report lists 17 new drugs for hypertension in clinical development in 2013 [3]. However, a closer look reveals that the prospects to see a new drug entering the rink are much less promising. From the 17 compounds in development, two deal with preeclampsia, two represent clinical studies of already approved drugs (aliskiren and azilsartan medoxomil) in pediatric population, and six are a fixed-dose combination of already established agents. There have been six novel molecules approved by the FDA for the treatment of hypertension in this millennium (valsartan in 2001, eplerenone in 2002, olmesartan in 2003, aliskiren in 2007, clevidipine in 2008, and azilsartan medoxomil in 2011). With other words, the last new first-in-class (maybe for some time the only in class) for hypertension, aliskiren, was approved 7 years ago, and the last new molecule, azilsartan, was approved 3 years ago. These numbers are in contrast with the large pipeline for heart failure which lists 30 drugs in development, or in contrast with the five novel anticoagulants (three different modes of action), four new antiplatelets (two different modes of action), or five new molecules for the treatment of pulmonary hypertension (four modes of action) having been approved in the same time period by the FDA [4].

The reluctance to invest in the development of novel molecules for hypertension can be explained by the highly competitive pharma-environment (several established drug classes, some with dozen of compounds, and a large number of generics) in the field of high blood pressure treatment. This situation prompted the interest in nonpharmacological means for high blood pressure reduction. Yet, after some promising trials and large hopes with nonpharmacological therapies, the previously recognized drivers for new treatment approaches still remain in place. We need to improve blood pressure control, deal with resistant hypertension, and we are still on the outlook for the “Holy Grail”: risk reduction beyond blood pressure reduction [5]. With these needs in mind, the prospect of seeing a new antihypertensive molecule to enter the scene might not look so gloomy. Although few, the compounds in development listed by PhRMA provide an exciting range of modes of action: neprilysin/neutral endopeptidase (NEP) inhibitor (alone/combined with an angiotensin (AT1) receptor blocker (ARB)), a phosphodiesterase 5 (PDE5) inhibitor, natriuretic agents, and a long-lasting vasointestinal peptide (VIP) analogue. Of course, the list is not all-inclusive. The discoveries of the “protective renin-angiotensin-aldosterone system (RAAS)” have triggered the interest in angiotensin AT2 receptor (AT2 receptor) agonists, angiotensin converting enzyme (ACE) 2 stimulators, or Mas receptor agonists. Although those are still in the preclinical phase, they are good candidates for a putative new antihypertensive in the near future.

In the present review, we are going to review the recent development and the therapeutic potential of these candidate molecules (Table 1).

**Table 1:** Molecules currently or previously in development for hypertension treatment

Mode of action	Compound(s) (phase of clinical investigation)
Investigation for hypertension (active/inactive)	
Angiotensin converting enzyme/neprilysin inhibitors	
Inactive	Sampatrilat (III), omapatrilat (III), ilepatril (AVE-7688, IIb/III)
Angiotensin (AT1) receptor/neprilysin inhibitors (ARNI)	
Active	LCZ696 (Sacubitril, AHU377) (III for HT and HF)
Inactive	VNP489 (I)
Endothelin receptor blockers	
Inactive	Darusentan (III), TBC3711 (II), Ambrisentan (2007 approved for PAH), Macitentan (2013 approved for PAH)
Endothelin receptor/angiotensin (AT1) receptor blockers	
Inactive	PS433540 (IIb)
Endothelin converting enzyme/neprilysin inhibitors	
Active	SLV336 (PC), SLV338 (PC), SLV306 (daglutril, KC126115) (II)
Phosphodiesterase 3 inhibitors	
Inactive	Cilostazol (1999 approved for IC)
Phosphodiesterase 5 inhibitors	
Active	KD027 (Slx-2101) (II)
Inactive	Vardenafil (2003 approved for PAH), Tadalafil (2009 approved for PAH; II for HT)
Vasoactive intestinal peptide analogue	
Active	PB1046 (II further studies probably in HF and PAH)
Natriuretic peptide and natriuretic molecules	
Active	PL3994 (IIa)
Inactive	MK-7145 (Ib), MK-8150 (Ib)
Angiotensin AT2 receptor agonists	
Active	Compound 21 (PC)
Inactive	LP2 (PC), CGP42112A (PC)
Mas receptor agonists	
Inactive	AVE-0991 (PC), NorLeu^3^-Ang (1–7) (PC), CGEN-856 (PC), PanCyte Ang (1–7) (PC)
Active	Hydroxypropyl-Ang (1–7) (PC)
Angiotensin converting enzyme 2 supplementation/activators	
Active	rhACE2 (APN01) (II further studies in acute lung injury)
Inactive	XNT (PC), diaminazene (DIZI) (PC)

*HF* heart failure, *HT* hypertension, *IC* intermittent claudication, *PAH* pulmonary arterial hypertension, *PC* pre-clinical phase

## Vasopeptidase Inhibitors

### Dual ACE/NEP Inhibition

NEP, neprilysin, or membrane metallo-endopeptidase is a metalloprotease which hydrolyses several peptide hormones rendering them inactive. Among its substrates are vasoconstritive (Ang I, II, endothelin) as well as vasodilative (natriuretic peptides, kinins) mediators [6]. The enhancement of natriuretic peptides concentrations by NEP inhibition was hypothesized to be able to augment the blood pressure-lowering properties of RAAS inhibition. The first dual inhibitors were combining NEP and ACE inhibition. Sampatrilat lowered blood pressure in hypertensive patients poorly controlled by ACE inhibitors [7]. Omapatrilat showed promising results not only in hypertensive patients in the OCTAVE trial but also in the heart failure OVERTURE trial as well. However, both of these trials reported higher occurrence of angioedema in patients treated with omapatrilat [8, 9]. Next-generation NEP/ACE inhibitor, ilepatril, (AVE7688) was designed to have improved specificity and prolong the ACE inhibition. Ilepatril dose-dependently reduced blood pressure in mild to moderate hypertensive patients in the phase IIb RAVEL-1 trial [10, 11]. Although a phase III trial was expected, no results were reported yet and no such trial is being listed on clinicaltrials.org.

### Dual Angiotensin (AT1) Receptor/NEP Inhibition

Another drug class combines NEP inhibition with an ARB moiety, so-called ARNI. LCZ696 combines the ARB valsartan moiety with the prodrug NEP inhibiting moiety sacubitril (AHU377). A breakthrough study for LCZ696 was its phase II, randomized, double-blind, placebo- and active-controlled clinical trial in 1328 patients with mild to moderate essential hypertension [12••]. After 8 weeks of treatment, 200 and 400 mg LCZ696 reduced sitting systolic and diastolic blood pressure more than the corresponding 160 and 320 mg valsartan doses. Moreover, 200 mg LCZ696 led to better blood pressure control and larger pulse pressure reduction compared to valsartan. Especially the safety results, which in contrast to the previous vasopeptidase inhibitors did not report any angioedema in this study [12••], have boosted further development of this compound. A smaller placebo-controlled study in 457 patients from Asia confirmed a significant blood pressure reduction after 8 weeks of treatment for 100, 200, and 400 mg LCZ696 and no occurrence of angioedema [13]. In addition, the effects of LCZ696 were investigated in the setting of heart failure or organ protection. First, LCZ696 achieved greater reduction in N-terminal pro b-type natriuretic peptide (NT-ProBNP) levels and preservation of estimated glomerular filtration rate compared to valsartan in the phase II PARAMOUNT trial in heart failure patients with preserved ejection fraction [14, 15]. Moreover, these effects of LCZ696 were independent of the systolic blood pressure reduction [16]. Second, the PARADIGM-HF trial in patients with heart failure with reduced ejection fraction was halted prematurely due to an overwhelming effect of LCZ696. Compared to enalapril treatment, LCZ696 reduced the primary end-point by 20 %, all-cause mortality by 16 %, and cardiovascular mortality by 20 % [17•]. In addition, LCZ696 reduced NT-ProBNP levels and slowed clinical progression more effectively than enalapril [18]. By indirect comparison, the all-cause mortality reduction translated to a striking 26–28 % reduction over putative placebo on the background of β-blocker and mineralocorticoid receptor antagonist (MRA) treatment [19]. In the PARADIGM-HF trial, angioedema developed in 10 out of 4187 patients in the LCZ696 group (compared to 5/4212 in the enalapril group), but there were fewer treatment discontinuations with LCZ696 compared to enalapril [17•]. Finally, beneficial effects of LCZ696 might comprise the attenuation of cardiac remodeling and dysfunction after myocardial infarction as suggested by a small animal study [20] or the improvement of aortic stiffness and central aortic hemodynamics as being currently investigated in the PARAMETER trial [21]. While LCZ696 is proceeding in clinical development for hypertension as well as heart failure [3], another putative ARNI, the VNP489, seems to be put on hold [22].

### Dual NEP/Endothelin Inhibition and Angiotensin (AT1) Receptor/Endothelin Blockade

Endothelin (predominantly endothelin-1, ET-1) via its receptors (ET_A_ and/or ET_B_) triggers vasoconstriction (both systemic as well as pulmonary), promotes inflammation, oxidative damage, fibrinogenesis, and atherosclerosis, and is involved in salt and water regulation [23–25]. The development of most studied endothelin receptor antagonist (selective ET_A_ antagonist, darusentan) for systemic hypertension was discontinued. Darusentan significantly reduced blood pressure in placebo- and active-controlled trials in hypertension DAR-311 (DORADO) and DAR-312 (DORADO-AC), but its tolerance was compromised by salt and water retention and the occurrence of peripheral edema [26, 27]. Another ET_A_ antagonist, TBC3711, which was previously investigated for resistant hypertension [5], has not been investigated further for the treatment of high systemic blood pressure. Several endothelin receptor antagonists are established in the treatment of pulmonary arterial hypertension. In this indication, even two new molecules, ambrisentan (2007) and macitentan (2013), have been approved by the FDA recently [4]. Their high selectivity for the pulmonary vasculature, which is desired in pulmonary hypertension treatment, renders them unlikely to be investigated for arterial hypertension.

Two different approaches were implemented in order to take advantage of ET-1 antagonism in the treatment of systemic hypertension: to combine ET-1 receptor antagonism with ARB or to combine endothelin converting enzyme (ECE) inhibition with NEP inhibition. For both of these approaches, some promising data were released, but both seem to be currently suspended. ARB/ET_A_ blockade was combined in PS433540, which in a phase IIb trial in stage 1–2 hypertensive patients reduced blood pressure compared to placebo and for the highest investigated dose (800 mg) also compared to irbesartan [22]. Dual ECE/NEP inhibition was combined in SLV306 (daglutril, prodrug for KC12615), SLV336, and SLV338. For SLV338, only preclinical data are available. It significantly reduced the incidence of stroke and improved survival in stroke-prone spontaneously hypertensive rats (spSHR), however, in a blood pressure-independent manner [28]. On the other hand, besides having reduced proteinuria and glomerulosclerosis in streptozotocin-induced diabetic rats similarly to captopril [29], daglutril attenuated pulmonary and right atrial pressure in patients with congestive heart failure [30]. The interest in daglutril might be revived by the recently published randomized, crossover, double-blind, placebo-controlled trial in hypertensive patients with type 2 diabetes and nephropathy. In this trial, daglutril improved blood pressure control and showed an acceptable safety profile. On the other hand, albuminuria in these patients remained unaffected by daglutril treatment [31, 32••].

## Phosphodiesterase Inhibition

Phosphodiesterases (PDE) inhibit the degradation of cyclic monophosphates. Of clinical significance are PDE3 and PDE5. The inhibition of PDE3 prevents the degradation of cyclic adenosine monophosphate (cAMP) and cyclic guanosine monophosphate (cGMP) preferentially in thrombocytes. A PDE3 inhibitor, cilostazol, is used to improve walking distance in patients with intermittent claudication. Cilostazol also attenuated pulmonary hypertension in rats [33] and improved right ventricle function and reduced pulmonary artery pressure in patients with right heart failure or moderate pulmonary arterial hypertension [34]. Due to its selectivity, cilostazol does not exert an influence on systemic blood pressure [35] and is not investigated for the treatment of hypertension. However, cilostazol reduces arterial compliance [35] and might improve cardiovascular risk in certain groups of patients [36, 37] suggesting some potential for its use as concomitant therapy in patients with arterial hypertension.

Even more research was focused on PDE5. PDE5 inhibitors reduce the degradation of cGMP, with the subsequent vasodilatory, antiproliferative, and antiaggregation effects. Due to the selective expression of PDE5, they have become established in the treatment of pulmonary arterial hypertension and erectile dysfunction. Since 2000, two new PDE5 inhibitors have joined sildenafil as approved drugs for pulmonary arterial hypertension: vardenafil (2003) and tadalafil (2009) [4]. Previously, sildenafil produced a 10/8 mmHg acute blood pressure reduction in a small study of six subjects with resistant hypertension. The effects were remarkably augmented by the combination of sildenafil with isomononitrate [38]. Tadalafil as well has previously been in the clinical phase II of investigation for arterial hypertension [5]. The blood pressure-lowering effect of tadalafil in the studies was low (−1.6/−0.8 mmHg) [39], and the drug has not been filed for approval in systemic hypertension. Nevertheless, in hypertensive patients, when tadalafil was added to metoprolol, bendrofluorothiazide, or an ARB (but not enalapril or amlodipine), it produced mild but significant blood pressure reduction [40]. Moreover, some recent studies suggested a positive, blood pressure-independent effect of tadalafil on left ventricular diastolic function in patients with resistant hypertension [41, 42]. Tadalafil is very unlikely to become a first- or second-line treatment for hypertension. Yet, due to its pleiotropic cardiovascular effects [43] in exceptional cases of resistant hypertension, its trial use might be warranted. Similar fate is likely to expect another PDE5 inhibitor, KD027 (Slx-2101). Slx-2101 is being investigated in phase II studies (NCT00562549, NCT00562614) for hypertension treatment, but no data on these trials have been published yet.

## Vasoactive Intestinal Peptide Agonist

Vasoactive intestinal peptide (VIP) is a neuropeptide hormone produced in many tissues, such as the intestine, pancreas, and hypothalamic nuclei. However, VIP potently modulates cardiovascular function as well. It stimulates contractility in the heart, causes vasodilation, increases glycogenolysis, lowers arterial blood pressure, and relaxes the smooth muscle of the trachea, stomach, and gall bladder. In humans, the VIP is encoded by the VIP gene. It shows vasodilation and positive inotropic properties via its vasoactive intestinal polypeptide receptors 1 and 2 (VPAC1 and VPAC2, respectively). The VIP levels were shown to be reduced in several models of hypertension and to correlate closely with left ventricular fibrosis [44]. However, the use of VIP in clinical situation is limited by its short half-life, low bioavailability, and VPAC1-mediated side effects. The VPAC2 selective, long-lasting VIP analogue, PB1046, enhanced myocardial contractility and reduced the demand of the myocardium in dogs [45•]. The vasodilation effects of PB1046 have been demonstrated in patients with essential hypertension. In two single-dose ascending studies (NCT01523067, NCT01873885), PB1046 was well-tolerated and produced a prolonged, dose-dependent effect on blood pressure [46]. In addition, further clinical development of the compound is planned for pulmonary hypertension and heart failure.

## Natriuretic Peptide Receptor A Agonists

Natriuretic peptides, such as the atrial and brain natriuretic peptides (ANP and BNP, respectively), provide natriuretic, vasodilatant, and antiproliferative effects via the natriuretic peptide receptor A (NPRA) and subsequent cGMP accumulation. Therefore, they might be considered for the treatment of hypertension, heart failure, nephrosclerosis, or stroke [47]. PL3994, MK-7145, and MK-8150 were reported to be in the clinical phase of development for hypertension [3]. PL3994 is a cyclic peptide ligand of the NPRA, which is however resistant to degradation by NEP [48]. PL3994 dose-dependently increased cGMP levels, reduced blood pressure, and induced natriuresis in healthy volunteers [49] and in patients with adequately controlled essential hypertension, in particular those treated with an ACE inhibitor [50]. While some development for PL3994 seems to continue, the results for a MK-7145 phase Ib study in hypertension (NCT01370655) are not available, and a study in heart failure patients (NCT01558674) was terminated. Similarly, two dosing studies (NCT01590810 and NCT01656408) were performed for MK-8150, but no results are available and neither of the Merck compounds is being listed in the Merck pipeline [51] anymore.

## The Protective RAAS

The inhibition of RAAS at various levels provides the current cornerstone for antihypertensive and cardioprotective therapies, such as ACE inhibitors, ARBs, renin inhibitors, mineralocorticoid receptor (MR) antagonists (MRAs), or even β-blockers [52]. However, besides the deleterious components of RAAS, such as ACE, Ang II, AT1 receptor, aldosterone, and the MR, there is also a “protective arm of the RAAS.” The backbone of the protective RAAS is represented by the effects of the AT2 receptor and Mas receptor stimulation. While the AT2 receptor is naturally stimulated by the Ang II, the inherent Mas receptor agonist is Ang (1–7) which is formed by cleavage of Ang II by ACE2 [53]. The discovery of these components has brought upon the concept of protective RAAS stimulation, which could supplement the inhibition of the deleterious arm of RAAS.

### Angiotensin AT2 Receptor Agonists

Unlike the AT1 receptor, the expression of the AT2 receptor is low in the adult vasculature but is upregulated in hypertension and vascular injury. The action of the AT2 receptor is partly opposing the AT1 receptor-mediated effects by triggering antiproliferation, regression of cardiovascular remodeling, and vasodilation [54]. The signaling pathways include the activation of protein phosphatases that inactivate the profibrotic mitogen-activated protein kinases (MAPKs) or the antiapoptotic Bcl-2 [55], NO/cGMP activation [56], and phospholipase A2 stimulation [57]. The first AT2 receptor agonists such as CGP42112A and LP2 were peptides, with lower specificity for AT2 receptor and not orally available. Therefore, the possibility to directly investigate the effects of direct pharmacological AT2 receptor stimulation has been fulfilled by the development of the first nonpeptide, orally available, specific, and selective AT2 receptor agonist, compound 21 [58].

The stimulation of the AT2 receptor does not produce vasodilation or blood pressure changes, unless the AT1 receptor is blocked as well [59]. Despite this fact, compound 21 might be useful in the condition of high blood pressure due to its immune modulatory properties. The stimulation of the AT2 receptor was demonstrated to lead to inhibition of nuclear factor κB (NF-κB) activity by epoxidation of 11,12-epoxyeicosatrienoic acid [60] with subsequent direct and indirect anti-inflammatory action with augmented interleukin (IL)-10 production [61, 62] and T cell differentiation to the *T*_reg_ phenotype [63].

It is being hypothesized that anti-inflammatory therapy, in particular IL-10 and transforming growth factor β (TGF-β) guided *T*_reg_-mediated immunosuppression, might provide an innovative strategy for the treatment of high blood pressure [64]. Compound 21 might represent a prototype and proof of this concept. Six-week treatment with compound 21 alone or in combination with an ARB was investigated in spSHR rats [65••] and L-N^ω^-Nitroarginine Methyl Ester (L-NAME)-induced hypertensive rats [66••]. In both studies, compound 21 reduced collagen content in the mesenteric arteries or in the aorta and improved the elastic properties of the vessels. The effect on the vascular wall properties elicited by compound 21 was comparable to the changes in the ARB-treated animals yet without any blood pressure effect. Moreover, when compound 21 was combined with an ARB, the collagen content was further reduced, without any additional significant blood pressure effect. It was postulated that de-stiffening strategies aimed at altering collagen and elastin balance and preventing premature aging are at the forefront of the search for target organ damage protection beyond the effects of blood pressure reduction. Such interventions could include the breaking of collagen cross-links or preventing their formation [67]. The available data on compound 21 suggest that it might confer such properties.

Compound 21 improved myocardial function after myocardial infarction in short-term [68] as well as extended [69•] treatment. These studies reported complex modulation of matrix metalloproteinase activities and collagen content via the modulation of TGF-β release. Selective AT2 receptor stimulation has demonstrated renoprotective effects in doxorubicin-induced chronic kidney disease [70], 2-kidney-1-clip hypertension [71], and in a high dose also in spSHR-fed high-salt diet [72]. In the kidneys, compound 21 affects the sodium/hydrogen exchanger 3 (NHE‑3) and the Na^+^/K^+^-ATPase in the proximal tubules, leading to natriuretic effects [73]. Other beneficial effects of compound 21 include the prevention of cognitive decline when added to *N*-methyl-d-aspartate (NMDA) receptors blockadein type II diabetic mice [74] and neuroprotective effects after spinal cord injury [75] or autoimmune encephalitis [63]. Compound 21 is currently undergoing the required toxicology studies to enter in the clinical phase of investigation.

### The ACE2/Ang (1–7)/Mas Receptor Axis Agonists

Another receptor belonging to the “protective RAAS” is the Mas receptor. Similarly to the AT2 receptor, the Mas receptor mediates effects such as antifibrosis, anti-inflammation, antiproliferation, or NO release. The blockade of either AT2 receptor or Mas receptor seems to block the effects of the other receptor, probably due to their hetero-dimerization [76••]. While the natural ligand for the AT2 receptor is Ang II, for the Mas receptor, it is the Ang (1–7). The possible strategies exploiting the Mas receptor stimulation include the development of peptide analogues, the protection of Ang (1–7), the development of a nonpeptide Mas receptor agonist, or to enhance endogenous Ang (1–7) production by recombinant ACE2 or by ACE2 activators [77].

The peptide Ang (1–7) analogues include NorLeu^3^-Ang 1–7, CGEN-856, and the cyclic Ang (1–7) analogue *PanCyte*. Most data for antihypertensive effects are available for CGEN-856, which dilated isolated aortic rings, reduced ischemia-reperfusion arrhythmias, and attenuated the blood pressure in spontaneously hypertensive rats [78]. The other peptide analogues were tested in conditions of pulmonary hypertension and pulmonary diseases.

To improve oral bioavailability, Ang (1–7) can be protected by hydroxyl-propyl‑β-cyclodextrin encapsulation. The encapsulated Ang (1–7) was recently shown to reduce blood pressure, heart rate, and myocardial hypertrophy in SHR [79••], inflammation in carotid atherosclerotic plaques [80], and ameliorated type 2 diabetes [81] in rats. It is expected to enter the clinical phase of development soon.

Very promising experimental data are available for the only available nonpeptide Mas receptor agonist, AVE-0991. In DOCA-salt-induced hypertension in rats, AVE-0991 did not only decreased mean arterial pressure when given alone but also when it was given on top of aliskiren treatment [82]. Besides blood pressure-lowering effects, AVE-0991 seems to exert blood pressure-independent renoprotective effects as well [83, 84]. Nevertheless, the development of AVE-0991 is currently suspended.

Finally, a mean to enhance Mas receptor stimulation is to increase endogenous Ang (1–7) levels by ACE2 supplementation or activation. The advantage of this approach is the simultaneous increase in Ang II degradation with subsequent attenuation of AT1 receptor stimulation.

Indeed, the spSHR have been shown to have reduced levels of ACE2. Moreover, the overexpression of ACE2 in these animals attenuated vasoconstriction, improved endothelium-dependent vasodilation, and reduced blood pressure [85]. This concept was tested in ACE2 knockout mice treated with Ang II infusion +/− recombinant human ACE2 (rhACE2). rhACE2 prevented cardiac remodeling including hypertrophy, myocardial fibrosis, increased procollagen I and II expression, TGF-β1, and fibronectin expression in Ang II-treated ACE2-knockout mice [86]. *Apeiron Biologics* brought rhACE, designated as APN01 (100 to 1200 μg/kg), to a phase 1 study in healthy volunteers. APN01 dose-dependently reduced the Ang II levels and increased Ang (1–7) and Ang (1–5) levels, the latter in a dose-dependent manner. On the other hand, there were no significant blood pressure or heart rate effects of ANP01 (except for small numeric reduction of systolic and diastolic blood pressure and heart after 800 and 1200 μg/kg ANP01 at the end of the infusion, which were only transient) [87••]. Thus, APN01 is unlikely to be investigated further for the treatment of high blood pressure. Instead, it is now licensed to *Glaxo-Smith-Kline* with the aim to enter a multicenter phase IIa study in patients with acute lung injury [77]. It was also hypothesized that rhACE2 might provide an interesting strategy for heart failure treatment [88].

Similarly to rhACE2, the stimulation of the endogenous ACE2 activity provides protective effects against target organ damage. In SHR, ACE2 activator, XNT, prevented renal [89] and myocardial [90] hydroxyproline accumulation. XNT prevented pulmonary vascular remodeling and right heart hypertrophy and fibrosis in monocrotaline-induced pulmonary hypertension [91]. The pharmacokinetic properties of XNT, however, are quite unfavorable [89]. Moreover, recent study demonstrated blood pressure-lowering effects of XNT but without any association with the modulation of plasmatic or renal ACE2 activity or Ang II breakdown ex vivo [92]. Therefore, it remains to be determined, whether XNT (or another ACE2 activator, diaminazene, DIZI) really affect ACE2 or whether they imply a different mechanism of action.

## Old Dogs, New Tricks

Despite the broad range of new possible therapeutic targets for hypertension, described above, it is apparently difficult to devise a new molecule that could be advanced to later phases of clinical investigation and that could successfully compete with the existing therapeutics. Nevertheless, there is a large opportunity to take the advantage of already broad choice of molecules and to optimize their usage.

### Drug Combinations

**Fig. 1:**
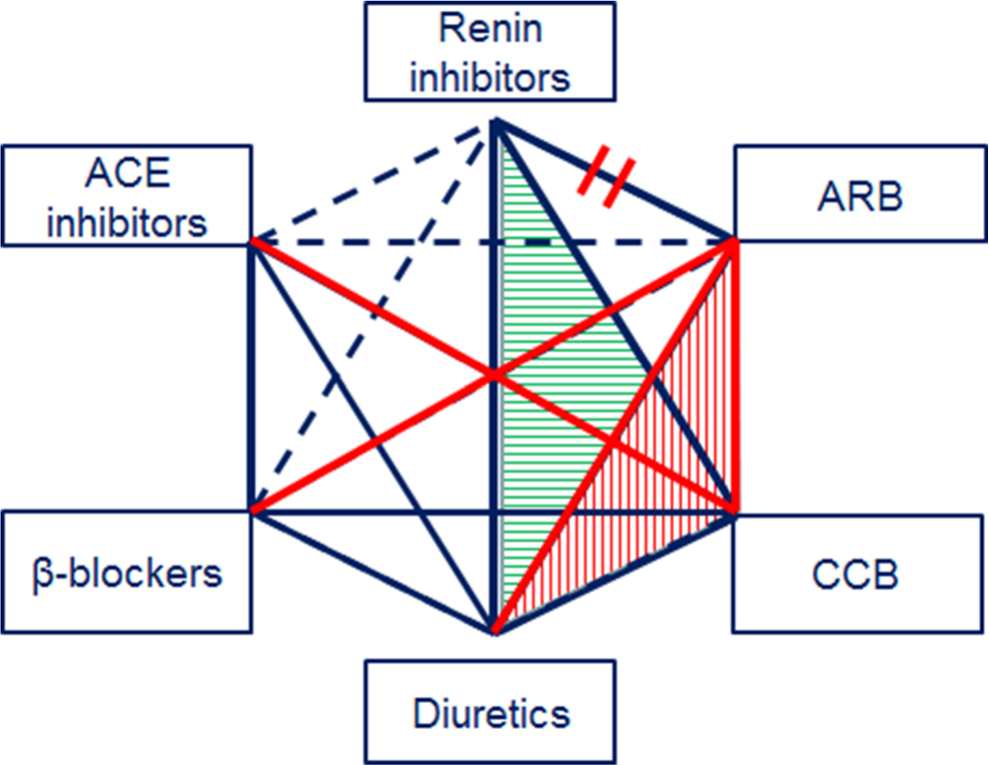
Recent development of fixed-dose combinations. Schematic representation adapting the combination hexagon from guidelines for the management of arterial hypertension [1] by including direct renin inhibitors (and omitting α-blockers). *Red lines* demonstrate combinations recently approved (since 2011) or in clinical phase of development in addition to the previously established combinations (*blue thick lines*). Aliskiren + valsartan combination was discontinued (*red double-crossed line*). *Patterned triangles* demonstrate approved triple therapies. *ARB* angiotensin (AT1) receptor blocker, *ACE* angiotensin converting enzyme, *CCB* calcium channel blocker

The use of combination therapy for the treatment of hypertension is already established in the practice and in the current guidelines [1]. Two or more antihypertensive molecules are combined together in the hope for superior blood pressure reduction or for the check-out of each other’s negative side effects (e.g., peripheral edema in calcium-channel blocker + ARB combination; or potassium loss in diuretic + ARB combination) [22]. The high number of possible combinations of antihypertensive drugs, some of them recommended, some without evidence, and some to be avoided, challenges the prescribing process for a thoughtful physician and complicates the adherence for the patient. Therefore, an increasing number of fixed-dose combinations are being introduced. Previously, we have reported that between 2000 and 2011, 10 new fixed-dosed double-combinations and three triple-combinations were approved were approved by the FDA [4, 22, 93]. Since then, two new double combinations (azilsartan medoxomil + chlorthalidone and perindopril + amlodipine) were approved [4] and five more (nebivolol + valsartan, amiloride + spironolactone, atorvastatin + losartan, amlodipine + losartan, candesartan + nifedipine) are pending [3] (Table 2). As previously expected [22], chlorthalidone (in addition to hydrochlorothiazide) is being introduced as a diuretic among these new combinations. When azilsartan was combined with chlorthalidone, its blood pressure-lowering effect and the achievement of target blood pressure levels was higher in comparison to fixed-dose combination with hydrochlorothiazide [94, 95]. A novel combination is the combination of an ARB + β-blocker, valsartan + nebivolol, adding to the available ACE inhibitor + β-blocker combination (lisinopril + carvedilol) (Fig. 1).

**Table 2:** Approved and clinically investigated fixed combinations for hypertension

Combination	
Drugs	Approved/investigated
Renin inhibitor + calcium channel blocker + diuretic	
Aliskiren + amlodipine + hydrochlorothiazide	Approved 2010
Angiotensin (AT1) receptor blocker + calcium channel blocker + diuretic	
Olmesartan + amlodipine + hydrochlorothiazide	Approved 2010
Valsartan + amlodipine + hydrochlorothiazide	Approved 2009
Renin Inhibitor + diuretic	
Aliskiren + hydrochlorothiazide	Approved 2008
Renin inhibitor + calcium channel blocker	
Aliskiren + amlodipine	Approved 2010
Renin inhibitor + angiotensin (AT1) receptor blocker	
Aliskiren + valsartan	Approved 2009, discount. 2012!
Angiotensin (AT1) receptor blocker + diuretic	
Azilsartan + chrothalidone	Approved 2011
Olmesartan + hydrochlorothiazide	Approved 2003
Eprosartan + hydrochlorothiazide	Approved 2001
Telmisartan + hydrochlorothiazide	Approved 2000
Valsartan + hydrochlorothiazide	Approved 1997
Angiotensin (AT1) receptor blocker + calcium channel blocker	
Losartan + amlodipine	Phase I
Candesartan + nifedipine	Phase III
Telmisartan + amlodipine	Approved 2009
Olmesartan + amlodipine	Approved 2007
Angiotensin (AT1) receptor blocker + β-blocker	
Valsartan + nebivolol	Phase III
Angiotensin converting enzyme inhibitor + diuretic	
Ramipril + hydrochlorothiazide	Approved 2009 (not available in the US)
Angiotensin converting enzyme inhibitor + calcium channel blocker	
Perindopril + amlodipine	Approved 2015
Enalapril + felodipine	Approved 1997
Enalapril + diltiazem	Approved 1996
Angiotensin converting enzyme inhibitor + β-blocker	
Lisinopril + carvedilol	Approved 2009 (not available in the US)
Angiotensin (AT1) receptor blocker + statin	
Losartan + atorvastatin	Phase II
Calcium channel blocker + statin	
Amlodipine + atorvastatin	Approved 2004
Diuretic combination	
Amiloride + spironolactone	Phase II

Based on [3, 4, 93]

From the numerous theoretically possible combinations, those blocking two steps within the RAAS should be avoided [1]. These recommendations are based on the results of the ALTITUDE and ONTARGET trials. In ALTITUDE, the addition of aliskiren to conventional antihypertensive treatment (an ACE inhibitor or an ARB) in patients with type 2 diabetes and renal impairment led to an increase in almost all primary end-point components (cardiovascular death, nonatal myocardial infarction, nonfatal stroke, resuscitated sudden death, doubling of serum creatinine, end stage renal disease/renal death) with exception of hospitalization for heart failure. The study was halted prematurely [96]. Based on the previous ONTARGET study results, dual RAAS inhibition with an ACE inhibitor + ARB is also not favorable in clinical conditions and should be discouraged [97]. With respect to preclinical studies, the result of the ALTITUDE study has been surprising; in particular, a renoprotective effect was expected from the aliskiren + ACE inhibitor/ARB combination. In fact, this combination therapy reduced proteinuria to a great extent. An increased incidence of hypotensive effects and renal adverse effects was likely reported due to the recruitment of high-risk patients with advanced renal injury and well-controlled blood pressure. The results of the ALTITUDE trial might have discouraged further development of other renin inhibitors. Several of them were previously in the pipelines, such as SPP635, SPP676, SPP1148, SPP1234, and VTP2799 [5]. New data were published only for VTP2799, which, however, has been shown to have different mode of action from aliskiren, with different pharmacokinetic and pharmacodynamic properties [98].

### Individually Tailored Therapy for Hypertension Based on Angiotensin Profile

Importantly, the ALTITUDE and ONTARGET results have also generated some hypotheses concerning the complex RAAS modulation by dual RAAS blockade. Single RAAS blockade by ACE inhibition or ARB leads to renin feedback resulting in the activation of the protective RAAS arm. Pharmacologic renin inhibition on top of ACE inhibition or ARB treatment might therefore abrogate the beneficial effects mediated by the alternative RAAS. The resulting decrease of the Ang (1–7)/Ang II ratio at the level of tissues may explain the increased incidence of adverse cardiovascular events after renin inhibition + ACE inhibition or ARB (but not after ARB + ACE inhibition) [99]. In fact, there are currently only sparse data on how antihypertensive monotherapy (e.g., ACE or renin inhibition) or even dual or triple therapies influence the levels of angiotensin peptides. The availability of novel and more reliable diagnostic tools for assessing the biochemical features of the RAAS might improve the understanding of patient-specific responses to RAAS inhibition [100]. Better understanding of the RAAS feedback mechanisms could open the doors not only for the development of novel drug combinations and therapeutic strategies but also most importantly for optimized personalized treatment schemes. The guidelines still somewhat represent a one-for-all approach. Studies should be aimed at identifying patient groups (high renin, high ACE, low ACE2 activity, etc.) that could mostly benefit from a particular treatment option (renin inhibitor, ACE inhibitor, ACE2 supplementation/activation, etc.). Such data could also help to differentiate different drugs among the current classes of hypertension therapies. For example, the most recent ARB, azilsartan medoxomil, increased Ang (1–7) levels and reduced renal 20-hydroxyeicosatetraenoic (HETE) acid levels along with prevention of hypertension and target organ damage in Ang II-induced hypertension in Sprague-Dawley rats [101]. In a meta-analysis of randomized active-controlled (comparators—ramipril, olmesartan, valsartan, candesartan, chlorthalidone) studies, azilsartan medoxomil conferred significantly higher reduction of office and ambulatory systolic as well as diastolic blood pressure than the comparators [102]. We may speculate, whether this superior blood pressure reduction was achieved due to the activation of the protective RAAS arm by azilsartan medoxomil. Alternate hypotheses could consider the pharmacologic profile of azilsartan medoxomil with slower AT1 receptor dissociation rates and higher receptor specificity [103], or its pleiotropic effects inhibiting endothelial cell proliferation and activating MAPKs in vascular smooth muscle cells [104]. Yet, there are no data, whether these blood pressure effects and additional mechanisms translate into morbidity or mortality effects (available only for valsartan, losartan a telmisartan). Further data on azilsartan medoxomil effects might be documented under clinical practice conditions in EARLY hypertension registry [105]. We also advocate for a hypertension registry that would prospectively follow patients with different RAAS profiles. Such data could provide some important clues to tackle the above-mentioned questions and open the door to individually tailored therapy based on particular patient profile.

### Aldosterone Antagonism

Another opportunity for individually guided therapy is the large prevalence of aldosteronism, in particular among resistant hypertensives [106, 107]. Such patients should be identified before being labeled resistant hypertensive and before nonpharmacological treatment is initiated. Instead, patients with high aldosterone levels could benefit from aldosterone antagonism. In 2002, eplerenone was added to spironolactone into the armamentarium of the MRAs. Its higher selectivity for MR and reduced affinity for sex steroid receptors resulted in better tolerability and less pronounced side effects. When added to conventional therapy, eplerenone reduced mortality and hospitalization rate in patients with systolic heart failure NYHA II in EMPHASIS-HF study [108] and reduced cardiovascular and all-cause mortality in patients after myocardial infarction with systolic heart failure in the EPHESUS study [109]. Next generation of MRAs, such as BAY94-8662, should provide higher selectivity for the MR compared with spironolactone and greater affinity compared with eplerenone [110]. In a phase II trial, BAY 94–8862 demonstrated tolerability and safety in patients with heart failure with reduced ejection fraction and mild or moderate chronic kidney disease. The markers of heart failure and chronic kidney disease were reduced similarly or more profoundly compared to spironolactone [111]. In addition to new MRAs development, aldosterone activity may be affected via the modulation of 11-β-hydroxysteroid dehydrogenase [112] or direct production inhibition.

Although MRAs are effective to reduce blood pressure, they can cause counter-regulatory increase in plasma renin and aldosterone levels, reducing treatment efficacy. Inhibition of aldosterone synthase (ASI) could reduce both MR-dependent (Na^+^/K^+^- or Na^+^/H^+^- pump activation) and MR-independent (protein kinase C or c-Jun N-terminal kinases activation) deleterious aldosterone effects, such as inflammation, vascular smooth muscle cell hypertrophy, vascular fibrosis, interstitial fibrosis of the kidney, and myocardial fibrosis and hypertrophy [5]. Several ASIs have been developed. LCI699 reduced ambulatory systolic blood pressures and plasma aldosterone levels in 14 patients with primary hyperaldosteronism [113] and decreased ambulatory blood pressure in patients with essential hypertension to comparable levels with 50 mg eplerenone twice daily (the highest approved dose) [114]. However, in patients with resistant hypertension, the blood pressure-lowering effects of LCI699 were inferior to eplerenone. Probably higher doses (which might bring upon off-target effects on cortisol synthesis) are required to achieve blood pressure reduction similar to eplerenone in this population [115]. Yet currently, the development of LCI669 is suspended similarly to a second generation of ASIs with improved selectivity (sparing the 11β-hydroxylase reaction), such as SPP2745 [116].

## Conclusion

From the present review, it is apparent that we should not be too much excited by the number of new molecules being claimed to be investigated for hypertension. With the high prevalence and clear primary read-out, hypertension is a good target for early drug development. Therefore, many new molecules enter early clinical phases in hypertension to establish their pharmacokinetic and pharmacodynamics profile. However, to find evidence which would distinguish a new substance from other antihypertensive drugs and which would establish this new molecule from marketing perspective is extremely difficult. Only morbidity/mortality data, which are hard and expensive to obtain, would make a true difference. Therefore, at the later stages, the development of most compounds is being suspended, interrupted, or shifted to commercially more perspective conditions such as heart failure or pulmonary hypertension. There is a dead end for hypertension treatment ahead.

Yet, there is still so much we do not know in hypertension. Actually, even for the established antihypertensives, we do not completely understand their mechanism of action. Even less, we really know about how to make the best use of the available drugs or what is the effect of the numerous possible double or triple combinations on endogenous vasomotive peptides and mediators. Obtaining such evidence could provide the real glimmer at the horizon in hypertension research. It would open the door for optimization of hypertension treatment, as well as for the development of new molecules. Some of them might be even reentering the antihypertensive arena after their label has been extended from heart failure or pulmonary hypertension treatment. We just should not give up and we should keep our eyes open.
